# Comfortable sleep monitoring: using physiological process interconnectedness during sleep for novel software sensors

**DOI:** 10.3389/fnetp.2025.1625947

**Published:** 2026-01-08

**Authors:** Anna Bavarsad, Elias August, Erna Sif Arnardóttir

**Affiliations:** 1 Reykjavik University Sleep Institute, Reykjavik University, Reykjavik, Iceland; 2 Department of Engineering, Reykjavik University, Reykjavik, Iceland

**Keywords:** heart rate, nasal/oral pneumo-flow, esophageal pressure, abdominal motion, thoracic motion, RIP belts, network physiology

## Abstract

**Introduction:**

Monitoring sleep-disordered breathing typically requires many sensors, including pneumoflow masks, measuring nasal and oral airflow, and esophageal pressure catheters. While these tools provide detailed information about airflow, effort, and respiratory mechanics, they can be uncomfortable, invasive, and less feasible for long-term, home-based, or large-scale sleep studies. In contrast, respiratory inductance plethysmography (RIP) belts offer a non-invasive and well-tolerated alternative.

**Methods:**

In this study, we introduce four models that estimate key physiological signals from either RIP-belt data or pneumoflow mask data. Specifically, we present a heart rate model based on the RIP-belt signal, a nasal pneumoflow model estimating airflow from the RIP-belt signal, and two esophageal pressure models – one based on the RIP-belt signal, and the other one based on pneumoflow mask data. Data from 55 participants with varying degrees of sleep-disordered breathing were analyzed.

**Results:**

When fitted to each participant individually, the heart rate model as well as the nasal pneumoflow model achieved a mean Pearson correlation of 0.60. The esophageal pressure model, using RIP-belt data, yielded a mean Pearson correlation of 0.65, while the model using pneumoflow mask data yielded a mean Pearson correlation of 0.52.

**Discussion:**

Although these models do not replace gold-standard instruments, they provide physiologically interpretable estimates from non-invasive inputs and demonstrate potential for scalable, lower-burden sleep monitoring, and highlight the potential of considering physiological interconnectedness to extract desired information. Future work will focus on further validation and clinical diagnostic utility.

## 1 Introduction

Sleep-disordered breathing (SDB) represents a significant public health challenge, given its strong association with cardiovascular morbidity and mortality (Phillipson, 1993). Accurate diagnosis of SDB typically requires monitoring nasal airflow, respiratory effort, and heart rate (HR), among other physiological signals. While in-laboratory polysomnography remains the gold standard, there has been a growing shift toward home sleep apnea testing (HSAT). However, even HSAT systems commonly rely on multiple sensors–including nasal/oral masks, electrocardiographic electrodes, and esophageal pressure monitors–which can cause discomfort and interfere with natural sleep (Collop et al., 2011; Jin and Sanchez-Sinencio, 2014; Iyer and Mhanna, 2016). Several reports highlight the limitations of current methods: nasal masks may provoke discomfort or panic, esophageal catheters are invasive and sensor displacement can compromise signal quality (Woodson and Wooten, 1992; Pepin et al., 2022; Zandieh et al., 2023). These limitations pose challenges for continuous, long-term, or ambulatory monitoring, particularly, in non-clinical settings. As a result, improving the comfort and usability of home-based diagnostic tools remains an important goal in sleep medicine. One promising direction is the use of respiratory inductance plethysmography (RIP) to capture thoracic and abdominal movements. RIP is a non-invasive, widely accepted technique for assessing respiratory effort, and it is currently recommended by the American Academy of Sleep Medicine (Berry et al., 2012). Compared to other motion sensors such as piezoelectric bands or accelerometers, RIP provides more stable signals and is less prone to artifacts (Butkov and Lee-Chiong, 2007).

From the early emergence of network physiology, the respiratory and cardiovascular systems have been viewed as dynamically coupled, with their coordinated behavior essential for maintaining physiological homeostasis (Bartsch et al., 2010; Bartsch et al., 2015). This coupling arises from both neural regulation and mechanical heart-lung interactions driven by intrathoracic pressure changes (Pinna et al., 2000; Grübler et al., 2017). Foundational studies have shown that physiological systems, such as the cardiac, respiratory, and neural one, form dynamic networks whose structures change across sleep stages (Bashan et al., 2012). Thus, our approach is motivated by the potential to extract multi-system information from a single physiological signal. Given the established physiological coupling between respiratory and cardiovascular systems, mediated through, both, neural and mechanical interactions, RIP signals may contain valuable information beyond respiratory effort (Bartsch et al., 2015; Pinna et al., 2000). This interdependence offers an opportunity to estimate additional parameters such as nasal airflow, HR, and esophageal pressure using only RIP-derived data.

In this study, we propose a novel software-based approach that uses RIP signals to estimate nasal airflow, heart rate, and esophageal pressure without requiring additional physical sensors. By reducing sensor burden, this approach aims to improve user comfort and data integrity in home-based SDB screening. Moreover, our analysis is based on complete overnight sleep recordings, typically spanning 10–11 h, allowing for a comprehensive evaluation of physiological patterns across the entire sleep period. The goal is not to replace gold-standard instrumentation, but to demonstrate the potential of RIP-based models to provide interpretable physiological estimates with reduced sensor burden. If validated more broadly, such approaches could enable more patient-friendly and cost-effective strategies for sleep disorder screening and monitoring, making large-scale and home-based SDB monitoring more feasible.

## 2 Materials and methods

We used full-night PSG data from 55 participants enrolled in the Sleep Revolution Project (Arnardottir et al., 2022). Of the 55 full-night PSG recordings, those deemed valid by the scorers, containing more than 4 h of usable data, were included in model training and evaluation. Therefore, 46 recordings were ultimately analyzed; that is, depending on quality of the signal under consideration between 39 and 44 were used. The mean age of participants was 39.5 ± 12.3 years, and 45% were female. The mean total sleep time (TST) was 365.7 ± 75.4 min, and the mean wake after sleep onset (WASO) was 80.2 ± 55.6 min. Complete participant characteristics, including gender, Body Mass Index (BMI), Apnea Hypopnea Index (AHI), Apnea Mixed, Apnea obstructed, and Hypopnea are provided in Supplementary Table S1 (Appendix). Ethical approval for this research was obtained from the National Bioethics Committee and the Data Protection Authority of Iceland under the reference VSN-21-082. Prior to participating in the study, all subjects were provided with detailed information about the research objectives and procedures, and they subsequently provided written informed consent. The PSG recordings were executed using A1 devices by Nox Medical in Reykjavik, Iceland. This allows the simultaneous acquisition of multiple physiological signals, through eight-lead electroencephalography, electromyography of the chin and leg, electrooculography, pulse oximetry (with a probe and sensor by Nonin, operating at a sampling rate of 75 Hz with a 1-s average for each pulse beat), a pneumo-mask for nasal and mouth breathing assessment (Glottal Enterprises, New York, United States), ECG, RIP belts on the chest and abdomen, a microphone strategically positioned on the chest, and actimetry coupled with positional registration, and an esophageal pressure measurement probe (MIKRO-CATH Pressure Catheter from Millar, Houston, United States).

### 2.1 Fourier transform

For a discrete-time signal x , the Discrete Fourier Transform is given by

$$X\left[k\right]=\sum_{n=0}^{N-1}x\left[n\right]e^{\frac{-j2\pi kn}{N}},$$ (1)

where $X\left[k\right]$ denotes the k -th Fourier coefficient and N is the number of samples in the signal. In practical applications, like sleep research, where signals are inherently discrete, the Fast Fourier Transform (FFT) efficiently transforms time-domain signals into their frequency-domain counterparts to extract frequency components for deciphering patterns within complex physiological signals (Oppenheim et al., 1997).

### 2.2 Statistical analysis

For assessing our regression models, we applied two complementary levels of statistical evaluation.

1. Model-level evaluation: We used the Root Mean Squared Error (RMSE) to quantify the average discrepancy between observed and predicted values, thereby evaluating predictive performance. Furthermore, we examined the R-squared and Adjusted R-squared coefficients to understand the proportion of variance in the dependent variable explained by the independent variables, with Adjusted R-squared providing a more accurate assessment by adjusting for the number of predictors in the model. We also evaluated the F-statistic against the constant model, along with its associated p-value, to determine the overall significance of the regression and whether the observed improvement in model fit is statistically significant.
2. Coefficient-level evaluation: For each regression coefficient, the standard error (SE) quantified estimation uncertainty, the t-statistic (tStat) tested the null hypothesis of no effect, and the p-value indicated statistical significance. Together, these measures allowed us to assess the strength and reliability of the relationships between independent and dependent variables, which is crucial for interpreting the impact of predictors on the outcome variable and for identifying statistically significant effects.

Note that the reported number of observations refers specifically to clean, artifact-free epochs included in the regression analysis for each subject, which represent a subset of the total recorded data after excluding segments affected by noise or signal dropout. The error degrees of freedom were defined as the number of valid epochs (observations) minus the number of estimated parameters.

### 2.3 Signal preprocessing and epoch definition

All physiological signals, including thoracic and abdominal effort, nasal pneumoflow, and esophageal pressure were first preprocessed to detect and remove artifacts such as signal dropout, sensor displacement, and high frequency noise. Segments affected by such artifacts were excluded prior to further analysis. The preprocessing procedure was tailored for each signal type and model according to its specific features and signal range, as detailed in the Methods section. After artifact removal, the clean signals were segmented into non-overlapping 60-s epochs. The number of samples in each epoch depended on the sampling rate of the specific model: For the first model with a 200 Hz sampling rate, each epoch contained 60 × 200 = 12,000 samples, whereas for subsequent models with a 250 Hz sampling rate, each epoch contained 60 × 250 = 15,000 samples. In this manuscript, the term “epoch” refers to these 60-s windows. The 60-s epoch length was chosen to provide enough samples for analysis, allow evaluation of cardiorespiratory features, and enable accurate FFT computation while retaining sensitive to rapid physiological changes. These epochs are also compatible with standard sleep scoring windows. In some figures, only a limited number of epochs may appear, due to recording duration and the length of artifact free signals.

### 2.4 Linear regression models and cross-validation

We used linear regression models for estimating desired physical variables. Model parameters were obtained using least-squares fitting, that is MATLAB’s “fitlm” function (MATLAB, 2023). First, we obtained model parameters using the entire data set comprising all participants. Model validation was conducted using a 10-fold cross-validation scheme, applied both across the pooled dataset of all participants and individually for each participant. In this approach, 90% of each participant’s data were used for training, while the remaining 10% were held out for testing. Performance was evaluated by calculating the Pearson correlation coefficient between predicted and observed values. We then repeated the procedure for each participant separately and summarized the results across the cohort.

### 2.5 Heart rate model based on respiratory effort

Our model is informed by Respiratory Sinus Arrhythmia (RSA), a phenomenon in which the heart rate changes with each breath (Giddens and Kitney, 1985); that is, the heart rate decreases during inhalation and increases during exhalation (Van Diest et al., 2014). Our model uses features related to breathing frequency and epoch duration to predict heart rate variations, capturing the significant relationship between respiration and heart rate.

Abdominal signals were sampled at 200 Hz and segmented into non-overlapping 60-s epochs. To improve data quality, noisy epochs were systematically excluded: pulse epochs exhibiting physiologically implausible heart rates (<40 or >180 bpm) or abrupt changes (>40 bpm between consecutive samples) were removed, alongside abdominal epochs exhibiting near-zero values indicative of sensor disconnection. Feature extraction was designed to capture physiologically relevant components of the abdominal signal. Frequency-domain features were obtained by applying the FFT to each epoch and computing the mean spectral magnitude within three distinct bands: low (0.07–0.3 Hz), mid (0.3–1.7 Hz), and high (1.7–5 Hz), corresponding to slow breathing dynamics, typical respiratory oscillations, and subtle cardiac-related thoracoabdominal fluctuations, respectively. These bands were selected to improve the physiological interpretability of the spectral information and to better capture interactions between respiration and cardiac activity.

To complement spectral descriptors and capture morphological variability, intercept and additional nonlinear features were computed. The root mean square (RMS) captured the overall signal power and intensity of respiratory effort. Skewness, a statistical measure of waveform asymmetry, was calculated for each epoch to quantify deviations from signal symmetry, providing insight into irregular or obstructed breathing patterns. These variables were included because variations in respiratory effort and waveform morphology can provide indirect yet physiologically meaningful cues about cardiac activity, particularly when standard pulse recordings are noisy or unavailable. The following linear regression model was fitted using the abdominal signals of all participants as independent variables to predict the average pulse per epoch from the combined spectral and nonlinear features,

$$pulse=\beta_{0}+\beta_{1}{\cdot X}_{band1}+\beta_{2}{\cdot X}_{band2}+\beta_{3}{\cdot X}_{band3}+\beta_{4}\cdot RMS+\beta_{5}\cdot Skewness,$$ (2)

where $\beta_{i}$ are coefficients estimated from the data, and $X_{bandi}$ denotes the mean spectral magnitude of the i -th frequency band extracted from the FFT of the abdominal signal for each epoch.

### 2.6 Nasal pneumo-flow model based on thoracic and abdominal movement

In this model, Nasal Pneumo-flow (NPF) is estimated from abdominal (AB) and thoracic (TH) RIP signals using the linear regression model shown below. After preprocessing, each signal was segmented into 60-s epochs, and epochs with artifacts or flat signals were removed. Then, for all *i*, we calculated the average spectral magnitude of the FFT of the AB and TH signals for epoch *i* and denoted them by $X_{ABi}$ and $X_{THi}$ , respectively, while the corresponding mean spectral magnitude of NPF was the target variable. The linear regression model is given by,

$$NPF=\beta_{0}+\beta_{1}{\cdot X}_{ABi}+\beta_{2}{\cdot X}_{THi}.$$ (3)

### 2.7 Esophageal pressure models

In this section, two models are proposed for estimating Esophageal Pressure (Pes), each relying on different physiological interconnectedness and, thus, using different signals to capture variations in breathing patterns and respiratory effort. Signals were preprocessed to remove artifacts and extreme outliers. Specifically, Pes spikes exceeding ±2 cmH_2_O and abrupt transitions were replaced using linear interpolation, followed by median filtering. Flat or corrupted epochs across all signals were identified and excluded, and the remaining data was segmented into equal-length epochs.

The first model $\left({P1}_{es}\right)$ combines NPF and Oral Pneumo-flow (OPF) to account for variations associated with both breathing modalities. The raw data from NPF, OPF, and Pes were divided into fixed-length (60s) epochs, where signal sampling frequency was 250 Hz. The FFT was applied to each signal to convert the time-domain data into frequency-domain features. For each channel, the mean spectral magnitude of the frequency was extracted, yielding a descriptor of signal energy. The nasal and oral mean spectral magnitudes, denoted by $X_{NPFi}$ and $X_{OPFi}$ for epoch i , served as predictors, while the corresponding mean spectral magnitude of the ${P1}_{es}$ signal, was the dependent variable. The regression model is given by,

$${P1}_{es}=\beta_{0}+\beta_{1}{\cdot X}_{NPFi}+\beta_{2}{\cdot X}_{OPFi}.$$ (4)

Differently to the previous approach, where nasal and oral pneumoflow signals were directly related to esophageal pressure, our second model $\left({P2}_{es}\right)$ uses thoracoabdominal motion to approximate pressure dynamics. For each epoch, the average spectral magnitude of the FFT was calculated for AB and TH signals (sampling frequency 200 Hz), serving as predictor variables, while the mean PES spectral magnitude was the target variable. The regression model is given by,

$$P2_{es}=\beta_{0}+\beta_{1}{\cdot X}_{ABi}+\beta_{2}{\cdot X}_{THi}.$$ (5)

**FIGURE 1:**
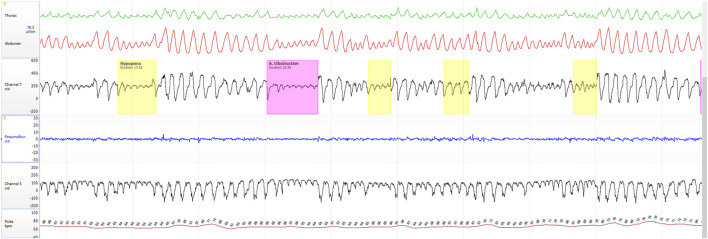
Representative 5-min segment from the same subject during an OSA event, showing aligned raw physiological signals: RIP (abdominal and thoracic effort), airflow (channel 7: Nasal Pneumoflow; Pneumoflow: Oral Pneumoflow), esophageal pressure (channel 5), and heart rate (Pulse).

**FIGURE 2:**
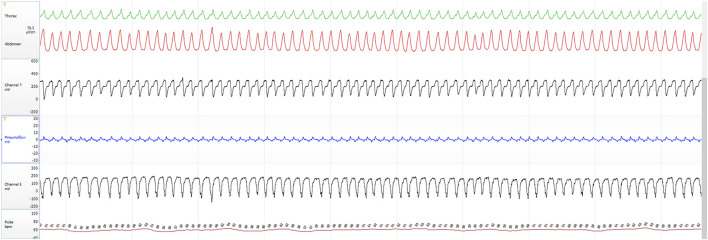
Five-minute aligned segment showing normal breathing from the same subject.

**FIGURE 3:**
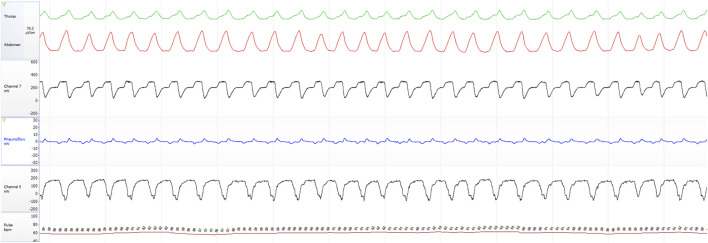
Two-minute aligned segment with a normal breathing from the same subject.

To provide a visual illustration of the relationship between input and output signals in the proposed models, Figures 1–3 show representative examples from the same individual during 2- and 5-min aligned segments. These include periods with obstructive sleep apnea (OSA) events as well as normal breathing. Each panel displays the raw, time-aligned physiological signals—RIP (abdominal and thoracic effort), airflow (nasal and oral), esophageal pressure (Pes), and heart rate (Pulse) — allowing direct visual comparison of respiratory patterns and model-derived outputs.

### 2.8 Preprocessing datasets

From the initial 55 recordings, nine were excluded because signal duration was below the threshold of 4 hours, leaving 46 valid datasets as described in Section 2.

#### 2.8.1 Heart rate model

For the HR model, three additional recordings were excluded: in one case the RIP belt was not attached, and in two cases the recordings exhibited a marked decline in RIP signal quality.

#### 2.8.2 Nasal pneumoflow model

For the Nasal Pneumoflow model, five additional recordings were excluded: Four participants stopped wearing the mask due to panic attacks and inability to breathe through it, their mask was replaced with a nasal cannula, or the mask moved during sleep. In addition, one did not have the RIP belt.

#### 2.8.3 Esophageal pressure models

For the esophageal pressure models, the same exclusion criteria applied to the input signals in the previous models (RIP belts or pneumoflow mask) were also considered. In addition, several recordings were excluded due to issues specific to the esophageal pressure signal itself, including premature termination of the study by the participant, missing or incomplete recordings, and contamination by cardiac-related artifacts when the catheter was positioned close to the heart. As a result, esophageal pressure model ${P1}_{es}$ was applied to a dataset comprising 39 participants and model ${P2}_{es}$ to a dataset comprising 44 participants.

## 3 Results

### 3.1 Fitting one model considering the entire dataset

We applied cross validation to assess generalizability. The comparable performance between the training and test sets indicated that the model generalizes well. Model performance was evaluated using the Pearson correlation coefficient (PCC) between predicted and observed pulse values (Table 1) and the RMSE.

**TABLE 1:** Performance of the four proposed models fitted using the entire dataset.

*Model*	*Number of participants*	*Mean PCC*	Std *PCC*	*Mean RMSE*	Std *RMSE*	*Number of observations*
HR	43	**0.286**	0.023	9.489	0.116	20,026
NPF	41	**0.184**	0.032	4.475	0.164	10,592
${P1}_{es}$	39	**0.348**	0.046	0.470	0.021	16,556
${P2}_{es}$	44	**0.437**	0.019	0.469	0.029	15,807

Bold values indicate the Pearson correlation coefficients (PCC) to highlight the model performance.

Model parameters and respective statistics for each of the four models are presented next. Tables 2A, 3A, 4A, 5A present the estimated regression coefficients for the four different models, taking the entire dataset into account. Tables 2B, 3B, 4B, 5B present respective standard errors, t-statistics, and p-values obtained when using the model with these parameters for prediction.

**TABLE 2A:** The HR model coefficients fitted using the entire dataset.

Coefficients	$\beta_{0}$	$\beta_{1}$	$\beta_{2}$	$\beta_{3}$	$\beta_{4}$	$\beta_{5}$
Estimates	68.7	95.2	30.4	833	−2.6e+05	−1.63

**TABLE 2b:** HR model parameters’ SE, t-Statistics, and p-values.

Predictor	SE	tStat	p-value
Intercept	0.170	404	0
Band 1 ( $X_{band1}$ )	3.5	28	6e-164
Band 2 ( $X_{band2}$ )	13.2	2.51	0.012
Band 3 ( $X_{band3}$ )	45.3	19.50	7e-84
RMS	11,584	−23	5e-115
Skewness	0.096	−16.6	3e-61

**TABLE 3a:** The nasal pneumoflow model coefficients fitted using the entire dataset.

Coefficients	$\beta_{0}$	$\beta_{1}$	$\beta_{2}$
Estimates	3.57	1305	2651

**TABLE 3B:** Nasal pneumoflow model parameters’ SE, t-Statistics, and p-values.

Predictor	SE	tStat	p-value
Intercept	0.081	43.4	0
$X_{ABi}$	272	5.24	2e-07
$X_{THi}$	289	9.41	6e-21

**TABLE 4a:** The ${P1}_{es}$ esophageal pressure model coefficients fitted using the entire dataset.

Coefficients	$\beta_{0}$	$\beta_{1}$	$\beta_{2}$
Estimates	0.52	0.041	0.063

**TABLE 4b:** The ${P1}_{es}$ Esophageal pressure parameters’ SE, t-Statistics, and p-values.

Predictor	SE	tStat	p-value
Intercept	0.0069	75.694	0
$X_{NPFi}$	0.0013	30.756	1e-226
$X_{OPFi}$	0.0023	27.107	4e-197

**TABLE 5a:** The ${P2}_{es}$ esophageal pressure model coefficients fitted using the entire dataset.

Coefficients	$\beta_{0}$	$\beta_{1}$	$\beta_{2}$
Estimates	0.47	41.1	867

**TABLE 5b:** The ${P2}_{es}$ Esophageal pressure parameters’ SE, t-Statistics, and p-values.

Predictor	SE	tStat	p-value
Intercept	0.0062	75.4	0
$X_{ABi}$	18.7	1.64	0.100
$X_{THi}$	22.5	39.0	2e-318

#### 3.1.1 Heart rate model

#### 3.1.2 Nasal pneumoflow model

#### 3.1.3 Esophageal pressure model

### 3.2 Fitting models using individual datasets

Table 6 presents PCC, RMSE, the R-squared and Adjusted R-Squared values, the F-statistic against the constant model, and the p-value obtained when fitting linear regression model parameters to each individual separately using only their dataset only and the using the model with these parameters for prediction.

**TABLE 6:** Performance of the four proposed models fitted for each participant individually.

Model	Mean PCC	Std PCC	Mean RMSE	Std RMSE	Mean number of observations	Mean error degrees of freedom	Mean R-squared	Mean adjusted R-squared	Mean F-statistic vs. constant model	Mean p-value
HR	**0.61**	0.09	4.15	0.54	414	396	0.375	0.365	42.9	6E-09
NPF	**0.60**	0.23	3.29	0.54	206	201	0.375	0.349	31.8	2.8E-03
${P1}_{es}$	**0.52**	0.17	0.39	0.107	347	344	0.290	0.287	104.0	4E-08
${P2}_{es}$	**0.65**	0.16	0.428	0.139	216	213	0.394	0.387	87.8	7.1E-05

**FIGURE 4:**
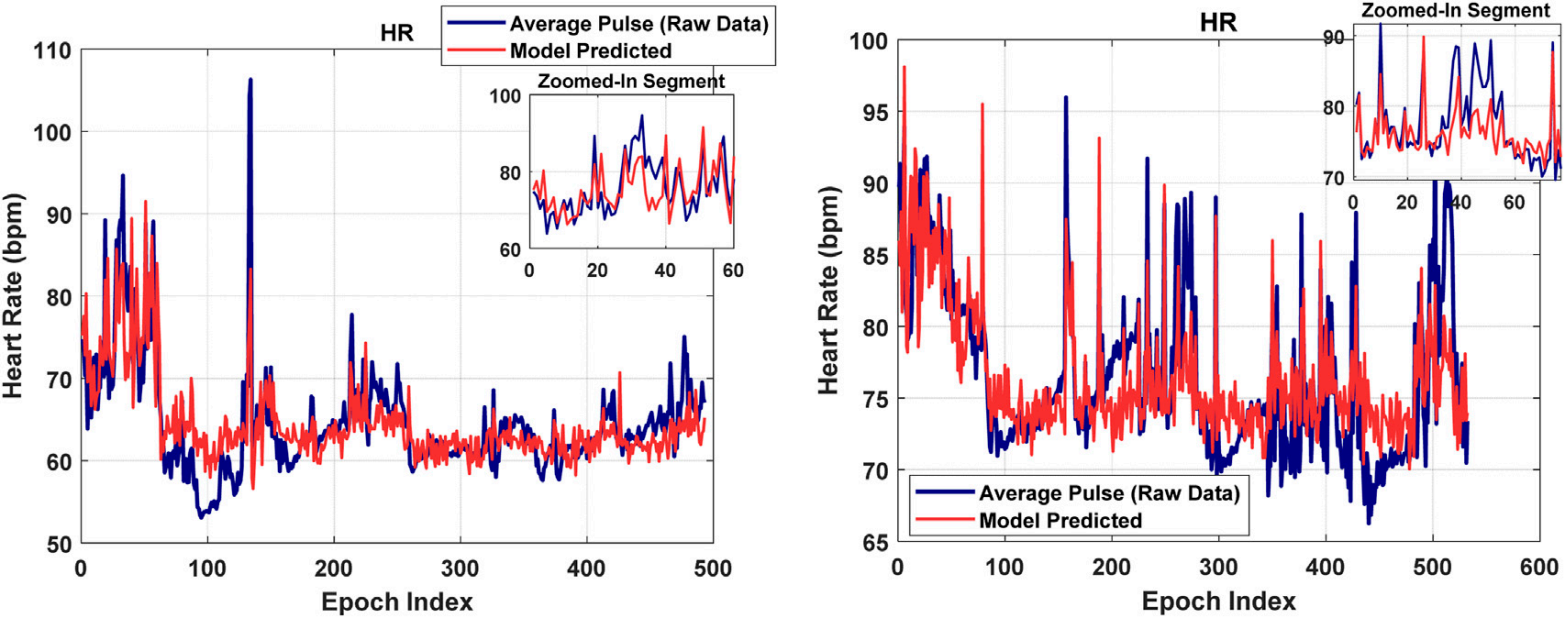
The performance of the model in estimating heart rate, shown for two participants.

**FIGURE 5:**
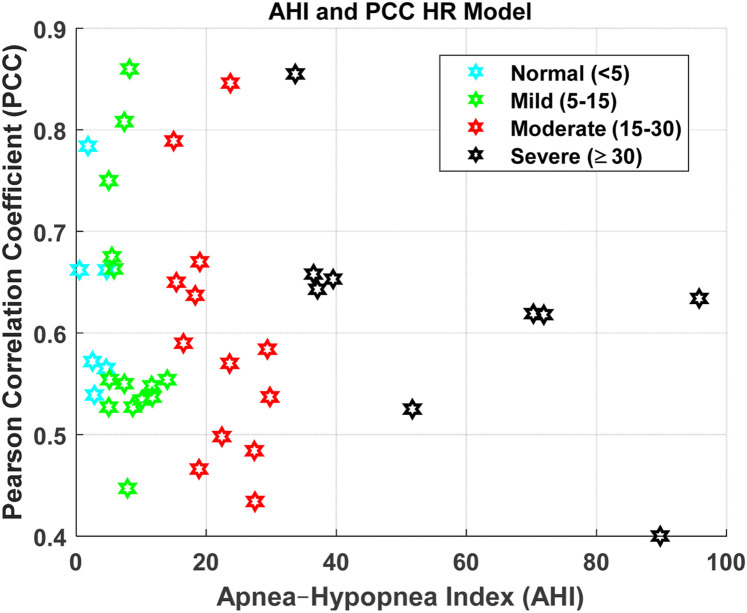
Impact of degree of participant’s AHI on the Pearson correlation coefficient for HR Model.

**FIGURE 6:**
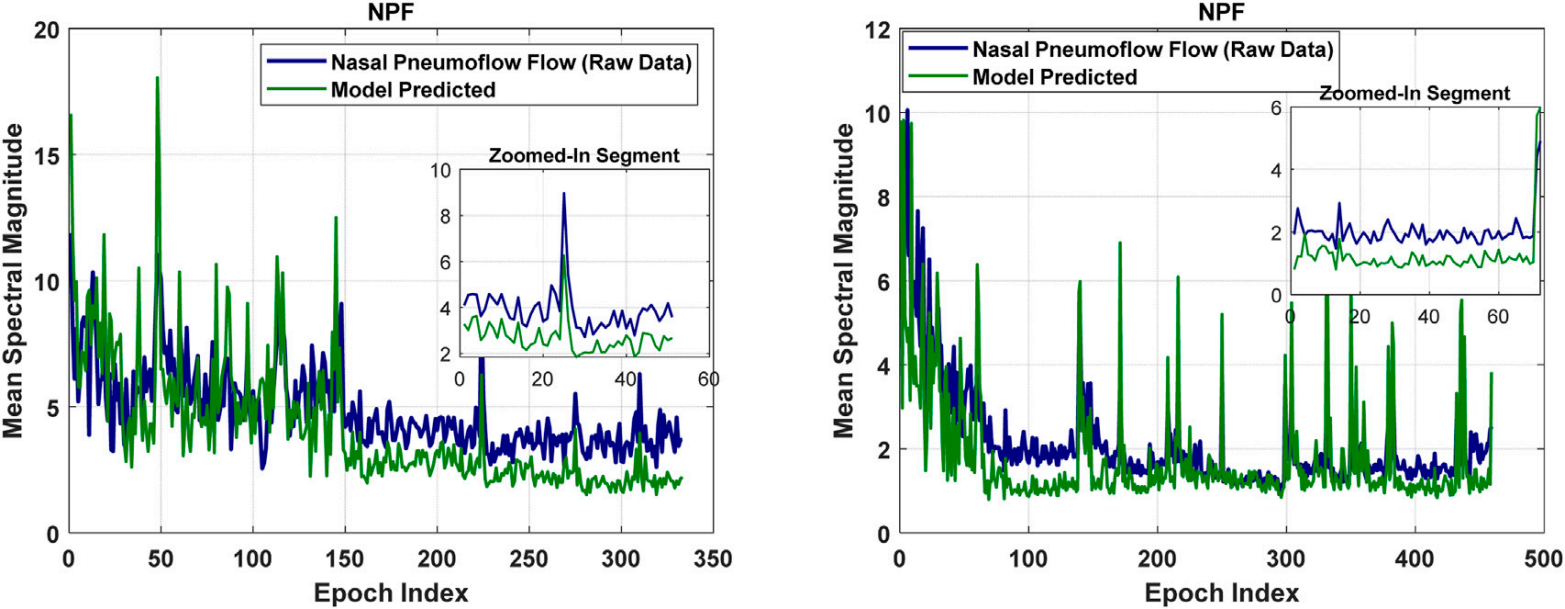
The performance of the model in estimating nasal pneumoflow, shown for two participants.

**FIGURE 7:**
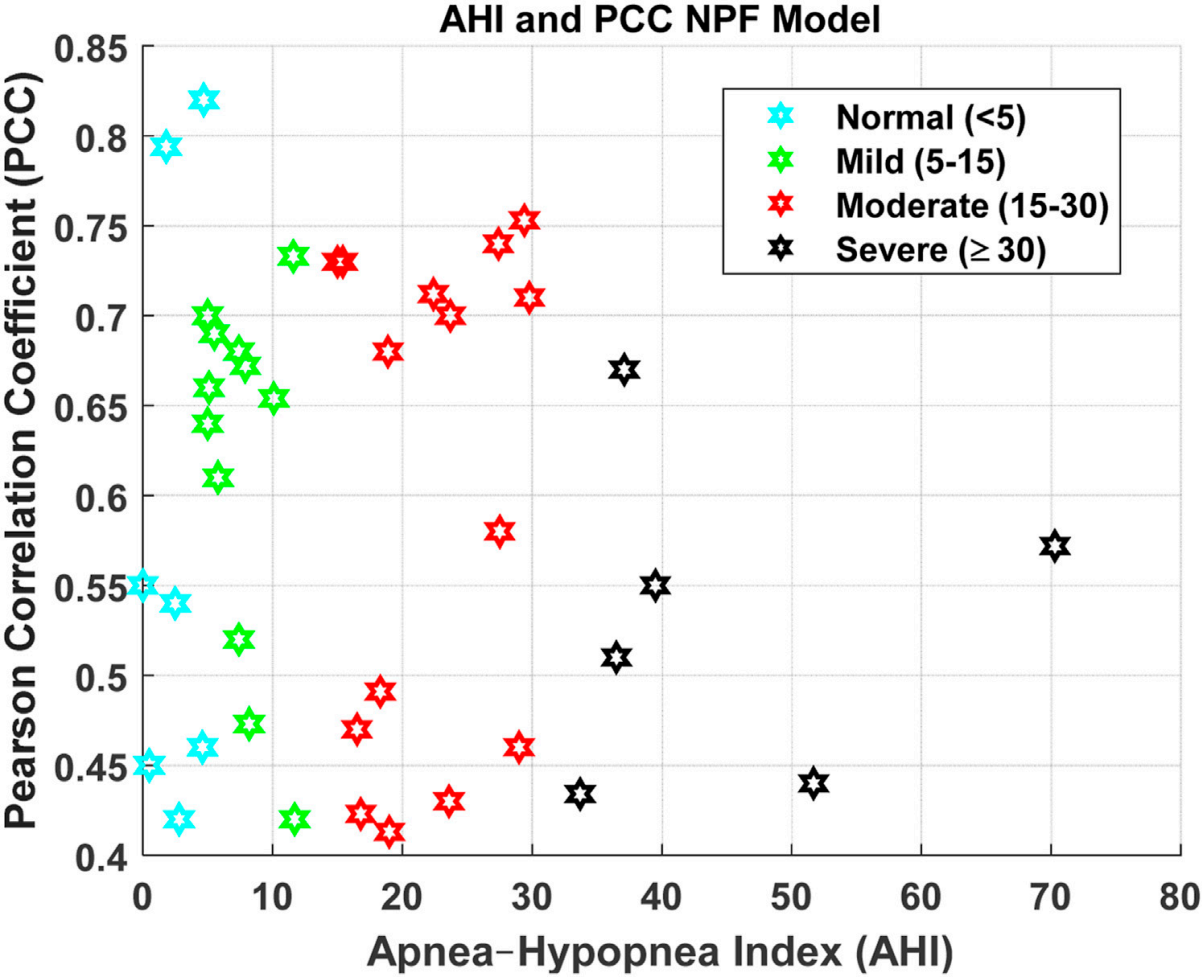
Analyzing the influence of different AHI levels on the Pearson correlation coefficient for the NPF model.

**FIGURE 8:**
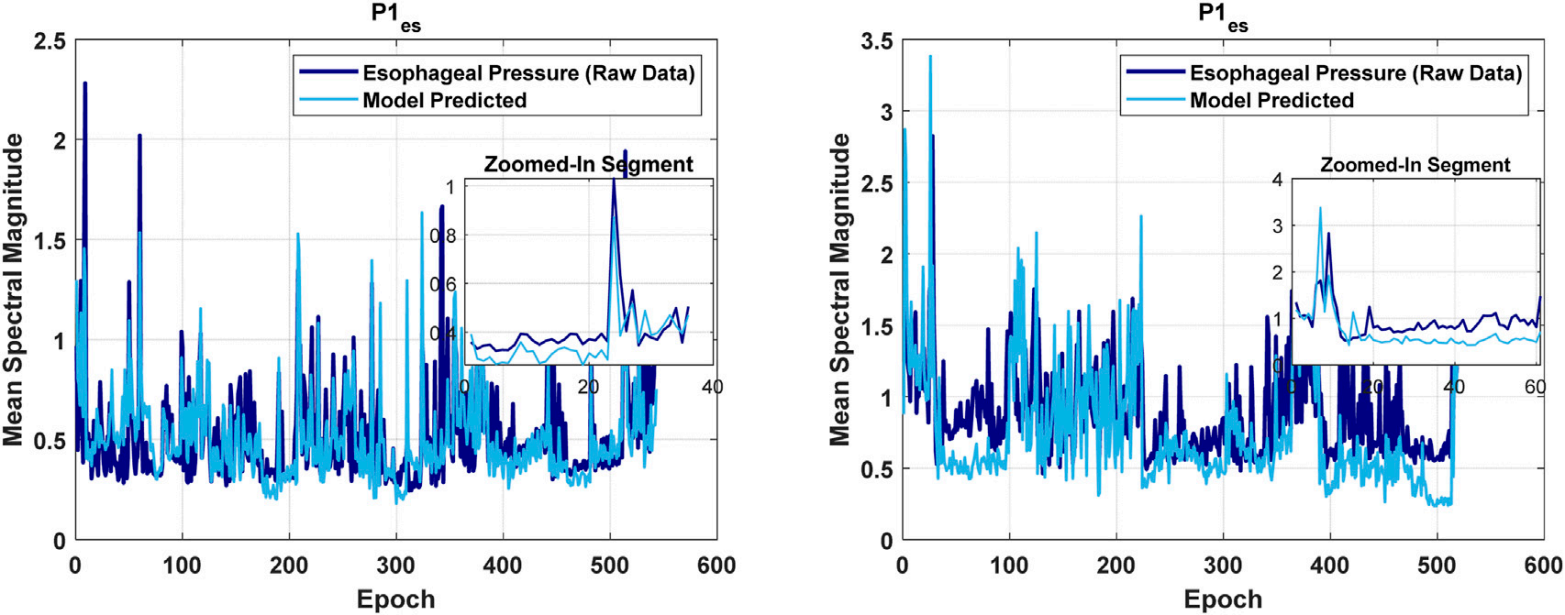
The Performance of the first model in estimating esophageal pressure.

**FIGURE 9:**
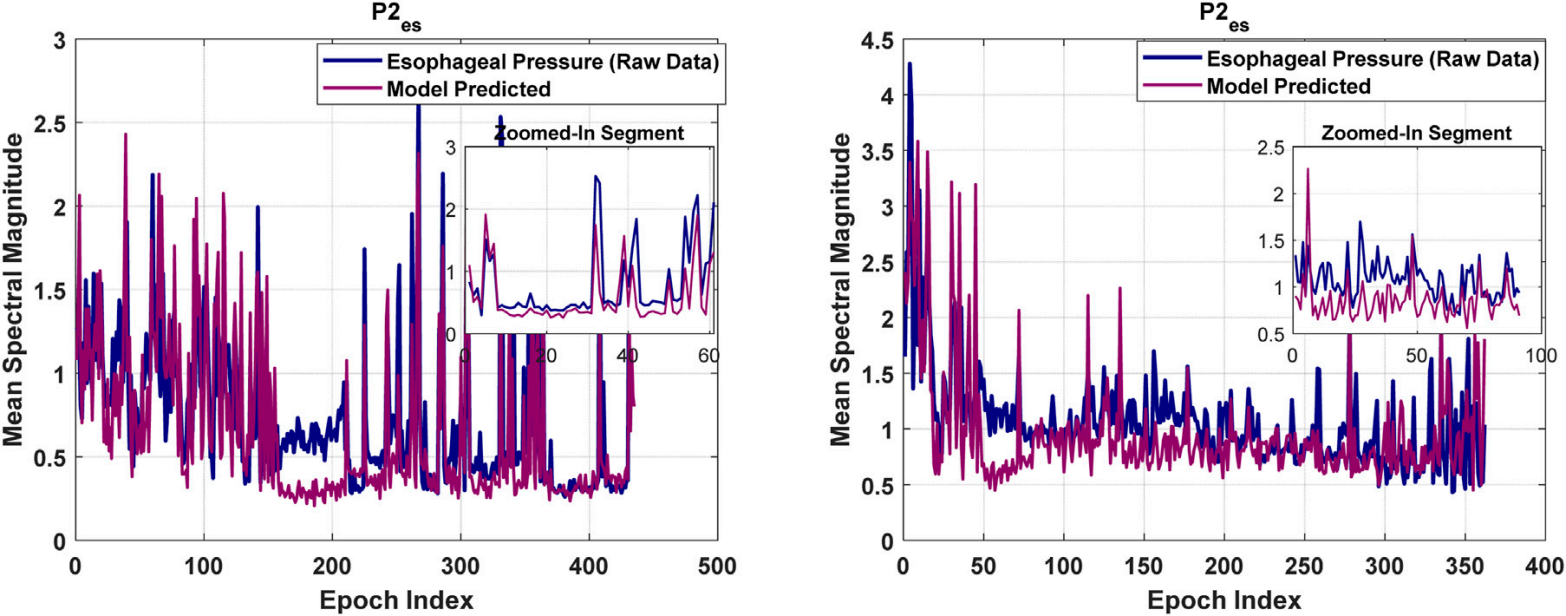
The second model’s efficacy in estimating esophageal pressure.

**FIGURE 10:**
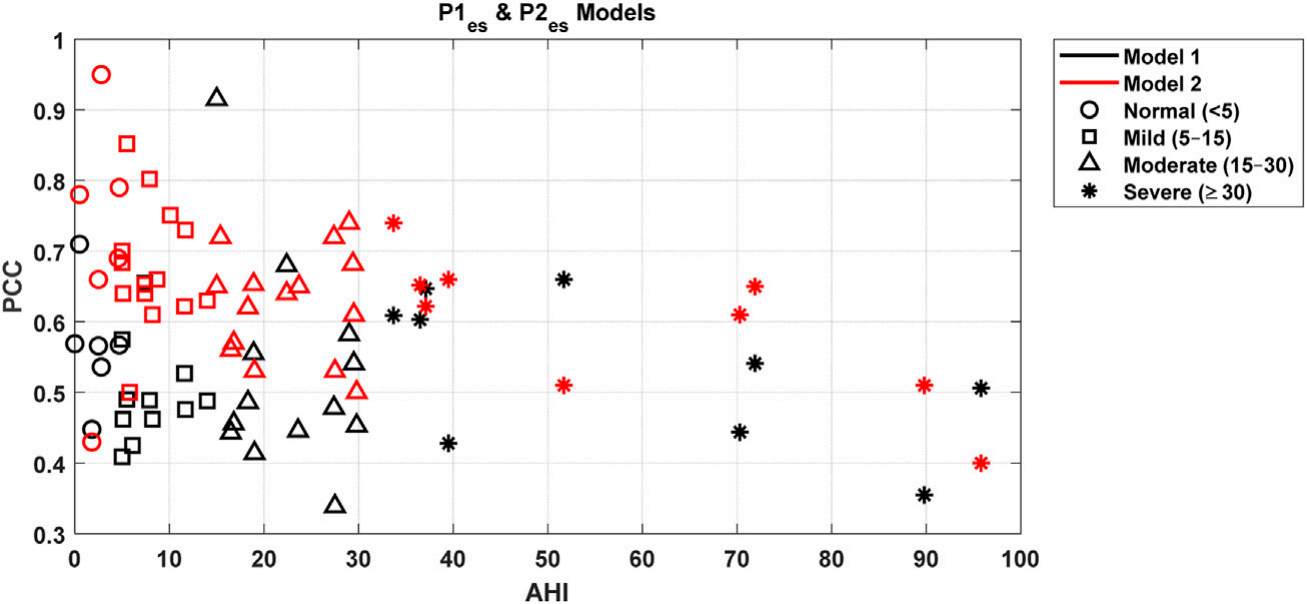
Impact of degree of AHI on the Pearson correlation coefficient for two esophageal pressure models.

**FIGURE 11:**
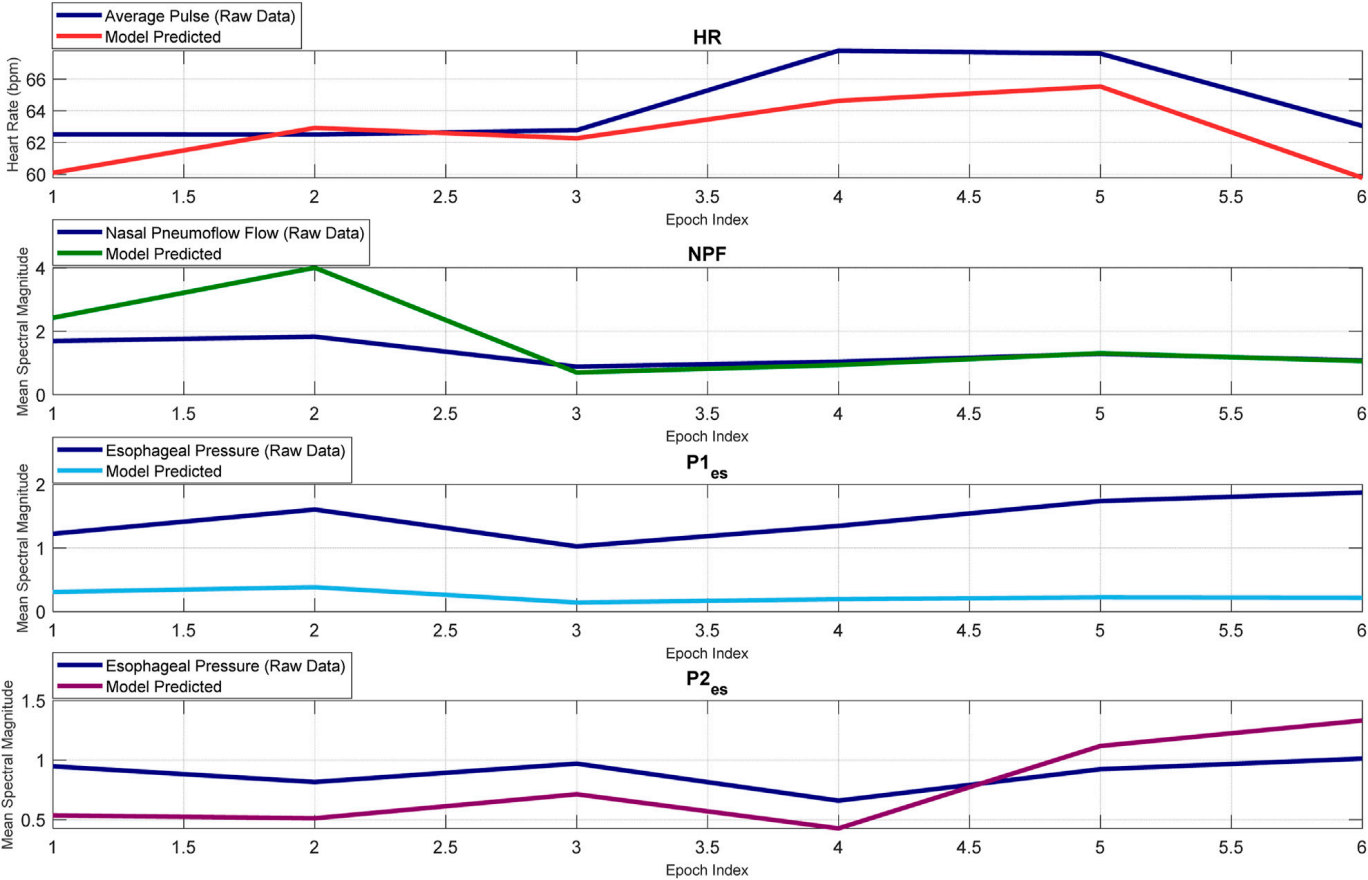
Synchronized outputs of four physiological models over a 5-minute segment.

Figures 4, 6, 8, 9 present illustrative cases for the four different models, showing the estimated and raw signals are plotted together to highlight the models’ performances. Figures 5, 7, 10 present the correlation coefficients obtained for different participants with different severities of AHI (normal, mild, moderate, or severe). Figure 11 illustrates the outputs of all four models vertically cascaded over a representative 5-min segment, providing a clear visual understanding of their temporal correspondence. This layout enables simultaneous inspection of the cardiac (Model 1), respiratory (Model 2), and esophageal pressure–related models (Models 3 and 4), revealing both well-aligned and weakly concordant periods across the physiological domains. It should be noted that minor temporal shifts may appear between some signals after preprocessing and artifact removal. For instance, a slight offset can be observed between the two esophageal pressure models. These adjustments were necessary to ensure that only valid, artifact-free segments were used for model training and comparison.

#### 3.2.1 Heart rate model

#### 3.2.2 Nasal pneumoflow model

#### 3.2.3 Esophageal pressure models

## 4 Discussion

Using the entire dataset to obtain a “one-fits-them-all” model, the correlation between dependent and independents variables is at best moderate. Nevertheless, in all four models, predictors show statistical significance, evident in the values of SE and tStat the low p-values. Thus, we interpret our first study as providing strong evidence for the proposed interplay of the different physiological systems that we exploit for the design of models for predicting important sleep related variables, particularly, as we propose relatively simple models. In our second study, when “calibrating” the proposed models for each individual separately by using only their dataset, we observe strong correlations between dependent and independents variables. Importantly, each dataset is rich, with an average of a couple hundred observations. Thus, such models or software sensors could eventually replace cumbersome sensors in home-based monitoring systems after calibration in a clinical setting. In the following, we further discuss the finding of our second study.

### 4.1 Heart rate model

The Pearson Correlation coefficient average is relatively high at 0.61. Thus, the model demonstrates strong predictive accuracy, successfully capturing overall trends in heart rate despite individual physiological variability and occasional mild movement artifacts during sleep. The model also demonstrates a moderate RMSE of 4.15, indicating reasonable predictive accuracy. The R-squared and Adjusted R-Squared values, averaging at 0.375 and 0.365 respectively, highlight our model’s ability to explain a significant portion of the output variable’s variance. Furthermore, the F-statistic against the constant model, averaging at 42.9, and the p-value of 6E-09 underscore the statistical significance of our model. These findings underscore the efficacy of our model in predicting heart rate. Notably, the model maintains consistent performance even under severe AHI conditions (Figure 5).

By leveraging frequency-selective spectral bands and complementary nonlinear features extracted from thoracic and abdominal RIP signals, the model provides a physiologically informed estimate of pulse-related activity, reflecting spectral activity in higher frequency bands where subtle ballistocardiographic components related to cardiac cycles may appear in thoracoabdominal signals. While it does not capture beat-to-beat variations, the model offers robust trend-level estimation suitable for further exploratory studies.

### 4.2 Nasal pneumoflow model

The model yields a mean Pearson correlation coefficient of 0.6, indicating a strong relationship between thoracic and abdominal signals and nasal pneumoflow. Notably, the model performs comparably well across varying apnea severities, supporting its applicability in diverse clinical contexts (Figure 7). By estimating mean spectral magnitude in the frequency domain, the model provides a physiologically grounded representation of respiratory effort and airflow magnitude.

The mean error degree of freedom is 201. The RMSE of 3.29 suggests that, on average, model predictions deviate from the actual values by this amount. The mean R-squared is 0.375, and the mean adjusted R-squared is 0.35. The latter accounts for the number of predictors in the model, suggesting that the model’s explanatory power remains robust even after considering the model’s complexity. Furthermore, the mean F-statistic of 31.83, with an associated mean p-value of 0.0028, indicates that the model’s outcomes are statistically significant.

### 4.3 Esophageal pressure models

Model 1: The mean Pearson correlation coefficient across participants is 0.52. Only for a few participants, the correlation coefficients are below the mean, typically reflecting segments of esophageal pressure data with notable fluctuations. The model’s RMSE is 0.39, indicating modest prediction error. The mean R-squared value is 0.29, with a mean adjusted R-squared of also 0.29, indicating that the model retains explanatory power even when accounting for the number of predictors. The mean F-statistic of 104, together with a mean p-value of 4E-08, confirms statistical significance.

Model 2: The mean Pearson correlation coefficient across all 44 participants is 0.65. Notably, for approximately 95% of participants (42 out of 44) correlation coefficients exceed 0.5. The mean error degrees of freedom is 213, reflecting sufficient variability to accurately estimate model parameters. The mean RMSE of 0.428 indicates modest prediction errors between the model’s outputs and the observed values. The mean R-squared value of 0.39 demonstrates that the model accounts for a meaningful portion of the variance in the outcome variable. The mean adjusted R-squared of also 0.39 confirms that the model’s explanatory power remains robust despite the inclusion of multiple predictors. Furthermore, the mean F-statistic of 87.77, along with the corresponding p-value of 7.1E-05, establishes statistical significance.

For the two proposed esophageal pressure models, model 2 exhibits better predictive accuracy with higher correlation coefficients, while model 1 shows more consistent results across participants. The superior performance of model 2 compared to model 1 can be attributed to differences in both signal acquisition modality and data quality. Model 1 relies on nasal and oral pneumoflow data, which were obtained using pneumoflow masks. These measurements are inherently more susceptible to artifacts due to mask displacement, participant discomfort, and movement during sleep, leading to data segments with reduced quality or missing information. In contrast, model 2 uses non-invasive thoracic and abdominal respiratory effort signals collected via RIP belts. This method is less intrusive and generally produces more stable and higher-quality signals across sleep epochs. Additionally, RIP belt recordings offer a more direct measure of thoracoabdominal motion, which physiologically correlates with esophageal pressure variations. The combination of improved signal quality and a more physiologically relevant input might thus explain the higher predictive accuracy of model 2.

### 4.4 Limitations

We have provided comprehensive statistical metrics across all proposed models (Table 6), including mean Pearson correlation, RMSE, *R* ^2^, adjusted *R* ^2^, F-statistics, and p-values. While these metrics demonstrate moderate-to-strong signal-level agreement, we emphasize that they do not establish diagnostic equivalence or clinical thresholds, which remain beyond the scope of this exploratory study. The current models are not intended as a diagnostic tool, but as an exploratory framework to inform future development of interpretable and low-complexity respiratory monitoring systems. Further important limitations and sources of potential bias that must be acknowledged are the following.

- Dataset size and diversity: The study includes only 55 participants, which limits the generalizability of the findings. This cohort may not fully represent the demographic and clinical variability present in broader populations, such as different ages, comorbidities, or ethnic backgrounds.
- Signal quality dependency: A major challenge across all models was the dependency on high-quality input signals. The RIP belt can be improperly positioned or loosened during sleep, introducing noise and reducing data reliability. Similarly, nasal mask displacement or discomfort may cause signal loss or artifacts in pneumo-flow data. These hardware-related issues significantly affect model accuracy and limit their robustness.
- Physiological variability: Individual differences in respiratory and cardiovascular physiology, as well as movement artifacts during sleep, introduce variability that the current models may not fully account for. This contributes to moderate rather than high predictive accuracy, as it highlights that despite generally acceptable performance, differences between individuals may necessitate a brief subject-specific calibration session to achieve optimal accuracy in real-world applications.
- Model complexity and assumptions: The models primarily rely on linear regression approaches and physiological assumptions that may not capture all nonlinear or complex interactions in the cardiorespiratory system during sleep. Additionally, the nasal pneumo-flow model operates solely in the frequency domain, estimating the mean spectral magnitude of respiratory signals. Although this approach effectively reflects relative respiratory effort, it does not reconstruct the airflow waveform in the time domain.
- Clinical applicability: While the models aim to reduce the number of sensors and improve patient comfort, further studies are needed to assess their diagnostic accuracy and practical utility in clinical workflows.

## 5 Conclusion and future work

In this paper, we presented novel models for predicting heart rate, nasal pneumo-flow, and esophageal pressure that make use of the strong interconnectedness of the respiratory network and cardiovascular network. We have evaluated their performance by comparing outcomes with raw data from a cohort of 55 participants. Based on the statistical results obtained for the four linear regression models, we inferr that these models demonstrate satisfactory capabilities in predicting the different sleep related variables. Notably, these models or software sensors, when further improved, will allow to reduce the necessity for multiple devices, effectively addressing concerns regarding patient discomfort and data integrity. Moreover, they highlight the potential that lies in investigating physiological networks for clinical use. The cohort of participants examined in our study presented a spectrum of sleep disorders, including obstructive and central apnea, as well as a combination of both (Supplementary Table S1 in the Appendix). In future investigations, we aim to delve deeper into the performance of our proposed models in diagnosing specific disorders and determining the AHI–a key metric in assessing the severity of sleep apnea. Therefore, in the future, we aim to also extend the impact of our proposed models beyond estimation towards reliable diagnosis and monitoring of sleep disorders, contributing to the ongoing evolution of effective and patient-friendly sleep health technologies.

Future work could also explore more precise spectral decomposition or adaptive filtering techniques to enhance temporal resolution and specificity, further linking thoracic and abdominal signal dynamics to cardiac physiology. Such developments could improve the Heart Rate Model’s sensitivity to beat-to-beat variations and expand its applicability in clinical and home-based monitoring environments. For the Nasal Pneumoflow Model, extending the approach to full-spectrum modeling and applying inverse FFT (IFFT) would enable accurate reconstruction of time-domain waveforms, facilitating detailed respiratory analysis. Importantly, the performance of this model should be further evaluated during specific respiratory events such as obstructive or central apneas, as these events involve altered physiology, particularly a dissociation between respiratory effort and airflow in OSA. Future studies should examine whether signal features such as paradoxical thoracoabdominal motion or waveform phase shifts can be leveraged to detect such events using the same non-invasive inputs. This event-level analysis was beyond the scope of the present study but remains an important direction for future investigation. Although we did not perform diagnostic thresholding or equivalence testing for the Esophageal Pressure Model, we encourage future investigations to build upon this framework and assess its clinical utility in more targeted diagnostic applications.

In summary, despite the limitations discussed in the previous section, the proposed models provide physiologically grounded, interpretable, and promising insights into the relationships between RIP signals, esophageal pressure catheters, and heart rate. While the models are not intended as diagnostic tools yet, they offer a valuable starting point for future research. Further refinement of the models, expanded datasets, and external validation are expected to enhance predictive accuracy and enable the development of interpretable, low-complexity monitoring systems. These results lay the foundation for future studies aiming to bridge RIP-derived signals with cardiopulmonary physiology under conditions closer to real-life sleep.

## Data Availability

The data analyzed in this study is subject to the following licenses/restrictions: This research is a part of the Sleep Revolution project, with funding from the European Union’s Horizon 2020 research and innovation program under grant agreement No. 965417. The data set can not be made available upon reasonable request - this is closed dataset.

## References

[B1] Arnardottir E. S. Islind A. S. Óskarsdóttir M. Ólafsdóttir K. A. August E. Jónasdóttir L. (2022). The sleep revolution project: the concept and objectives. J. Sleep. Res. 31, e13630. 10.1111/jsr.13630 35770626

[B2] Bartsch R. P. Liu K. K. Ma Q. D. Ivanov P. C. (2010). “Three independent forms of cardio-respiratory coupling: transitions across sleep stages.” in Computing in Cardiology. IEEE. Comput. Cardiol. 781–784. PMC4319215 25664348

[B3] Bartsch R. P. Liu K. K. L. Bashan A. Ivanov P. C. (2015). Network physiology: how organ systems dynamically interact. PLoS ONE 10 (11), e0142143. 10.1371/journal.pone.0142143 26555073 PMC4640580

[B4] Bashan A. Bartsch R. P. Kantelhardt J. W. Havlin S. Ivanov P. C. (2012). Network physiology reveals relations between network topology and physiological function. Nat. Commun. 3 (1), 702. 10.1038/ncomms1705 22426223 PMC3518900

[B5] Berry R. B. Budhiraja R. Gottlieb D. J. Gozal D. Iber C. Kapur V. K. (2012). Rules for scoring respiratory events in sleep: update of the 2007 AASM manual for the scoring of sleep and associated events: deliberations of the sleep apnea definitions task force of the American academy of sleep Medicine. J. Clin. Sleep Med. 8 (5), 597–619. 10.5664/jcsm.2172 23066376 PMC3459210

[B6] Butkov N. Lee-Chiong T. L. (2007). Fundamentals of sleep technology (Lippincott Williams & Wilkins).

[B7] Collop N. A. Tracy S. L. Kapur V. Mehra R. Kuhlmann D. Fleishman S. A. (2011). Obstructive sleep apnea devices for out-of-center (OOC) testing: technology evaluation. J. Clin. Sleep Med. 7 (5), 531–548. 10.5664/JCSM.1328 22003351 PMC3190855

[B8] Giddens D. P. Kitney R. I. (1985). Neonatal heart rate variability and its relation to respiration. J. Theor. Biol. 113 (4), 759–780. 10.1016/s0022-5193(85)80192-2 4033152

[B9] Grübler M. R. Wigger O. Berger D. Bloechlinger S. (2017). Basic concepts of heart-lung interactions during mechanical ventilation. Swiss Med. Wkly. 147, w14491. 10.4414/smw.2017.14491 28944931

[B10] Iyer N. P. Mhanna M. J. (2016). Association between high-flow nasal cannula and end-expiratory esophageal pressures in premature infants. Respir. care 61 (3), 285–290. 10.4187/respcare.04317 26508770

[B11] Jin J. Sánchez-Sinencio E. (2014). A home sleep apnea screening device with time-domain signal processing and autonomous scoring capability. IEEE Trans. Biomed. Circuits Syst. 9 (1), 96–104. 10.1109/TBCAS.2014.2314301 25486649

[B20] MATLAB (2023). MATLAB version: 23.2.0.2485118 (R2023b). Natick, Massachusetts: The MathWorks Inc. Available online at: https://www.mathworks.com .

[B12] Oppenheim A. V. Willsky A. S. Nawab S. H. (1997). Signals & systems. Pearson educación.

[B13] Pepin J. L. Le-Dong N. N. Cuthbert V. Coumans N. Tamisier R. Malhotra A. (2022). “Mandibular movements are a reliable noninvasive alternative to esophageal pressure for measuring respiratory effort in patients with sleep apnea syndrome,”. Nat. Sci. Sleep. 635–644. 10.2147/NSS.S346229 PMC9013709 35444480

[B14] Phillipson E. A. (1993). Sleep apnea-a major public health problem. N. Engl. J. Med. 328 (17), 1271–1273. 10.1056/NEJM199304293281712 8464440

[B15] Pinna G. D. Maestri R. Mortara A. La Rovere M. T. (2000). Cardiorespiratory interactions during periodic breathing in awake chronic heart failure patients. Am. J. Physiol. Heart Circ. Physiol. 278, H932–H941. 10.1152/ajpheart.2000.278.3.H932 10710362

[B16] Van Diest I. Verstappen K. Aubert A. E. Widjaja D. Vansteenwegen D. Vlemincx E. (2014). Inhalation/exhalation ratio modulates the effect of slow breathing on heart rate variability and relaxation. Appl. Psychophysiol. Biofeedback 39, 171–180. 10.1007/s10484-014-9253-x 25156003

[B18] Woodson B. T. Wooten M. R. (1992). A multisensor solid‐state pressure manometer to identify the level of collapse in obstructive sleep apnea. Otolaryngology-Head Neck Surg. 107 (5), 651–656. 10.1177/019459989210700507 1437203

[B19] Zandieh S. Kirschenbaum M. A. Greenberg H. Ancoli-Israel S. (2023). Keep it simple: a novel technique for measuring airflow using a wireless patch. Sleep. Health 9 (1), 100–107. 10.1016/j.sleh.2022.10.005 36473786

