# Supplementary material for: Comfortable sleep monitoring: using physiological process interconnectedness during sleep for novel software sensors
Source: Front Netw Physiol. 2026 Jan 8;5:1625947. doi: 10.3389/fnetp.2025.1625947 (PMC12823957; doi:10.3389/fnetp.2025.1625947)
Supplement: Supplementary file 1 [file Supplementaryfile1.docx]

# Appendix

Supplementary Table 1. Demographic, clinical, and sleep characteristics of participants, including BMI, sleep apnea status, and AHI.

| Patients | Status | TST (m) | WASO | Sleep Total Sleep Time | Age (years) | Gender (Female (F) & Male (M)) | BMI | AHI | Apnea Mixed | Apnea 0bstructed | Hypopnea |
| --- | --- | --- | --- | --- | --- | --- | --- | --- | --- | --- | --- |
|  | Scored | 318 | 54 | 5h 18m | 31 | F | 24.36 | 2.8 | 0 | 1 | 15 |
|  | Scored | 400.3 | 29.7 | 6h 40m | 24 | M | 25.96 | 15 | 1 | 4 | 40 |
|  | Scored | 426.5 | 48.5 | 7h 6m | 41 | F | 37.53 | 8.7 | 0 | 2 | 62 |
|  | Invalid<4h | - | - | - | 43 | M | 28.46 | 68.2 | 0 | 147 | 75 |
|  | Scored | 426.5 | 33.5 | 7h 6m | 21 | F | 19.51 | 1.8 | 0 | 0 | 14 |
|  | Scored | 403 | 21.5 | 6h 43m | 48 | M | 32.12 | 23.6 | 0 | 13 | 146 |
|  | Invalid | - | - | - | 39 | M | 37.2 | 23.9 | 0 | 0 | 0 |
|  | Scored | 322.5 | 129.9 | 5h 22m | - | M | 26.49 | 36.7 | 0 | 18 | 205 |
|  | Invalid | - | - | - | 51 | M | 30.43 | 1.3 | 0 | 0 | 0 |
|  | Scored | 334.3 | 77.6 | 5h 34m | 32 | F | 41.09 | 29.8 | 0 | 35 | 138 |
|  | Scored | 275.5 | 34.6 | 4h 17m | 48 | F | 35.43 | 7.9 | 0 | 2 | 35 |
|  | Scored | 302 | 130.5 | 5h 2m | - | - | 24.27 | 7.4 | 0 | 1 | 40 |
|  | Scored | 412 | 72.7 | 6h 52m | 36 | M | 23.99 | 7.4 | 3 | 1 | 51 |
|  | Scored | 242.5 | 148.1 | 4h 2m | 41 | M | 28.64 | 29.4 | 14 | 35 | 72 |
|  | Scored | 343 | 61.4 | 5h 43m | - | - | 34.76 | 70.3 | 12 | 47 | 170 |
|  | Scored | 336.5 | 89.7 | 5h 36m | 48 | M | 30.34 | 95.8 | 153 | 34 | 113 |
|  | Invalid<4h | - | - | - | 46 | M | 35.37 | 6.1 | 0 | 0 | 0 |
|  | Scored | 333.5 | 168.8 | 5h 33m | 50 | M | 32.28 | 29.5 | 0 | 10 | 143 |
|  | Scored | 312 | 151.5 | 5h 12m | 43 | F | 22.25 | 1.2 | 0 | 0 | 5 |
|  | Scored | 390.5 | 39.8 | 6h 30m | 28 | F | 26.02 | 5.5 | 0 | 0 | 33 |
|  | Invalid<4h | - | - | - | - | - | 43.56 | 120.0 | 4 | 12 | 51 |
|  | Scored | 264 | 146.5 | 4h 24m | 66 | M | 24.65 | 39.5 | 1 | 26 | 116 |
|  | Scored | 435.5 | 86.1 | 7h 15m | - | - | 33.33 | 5.1 | 0 | 1 | 43 |
|  | Scored | 472 | 43.3 | 7h 52m | 59 | F | 28.37 | 5.8 | 0 | 0 | 50 |
|  | Scored | 377 | 130.5 | 6h 17m | 49 | M | 25.63 | 18.9 | 0 | 12 | 114 |
|  | Scored | 277.9 | 130 | 4h 37m | 58 | M | 24.34 | 18.3 | 0 | 13 | 76 |
|  | Scored | 267 | 50.8 | 4h 27m | 61 | F | 24.25 | 10.1 | 1 | 0 | 51 |
|  | Scored | 349.2 | 155.3 | 5h 49m | 47 | F | 25.84 | 23.7 | 0 | 3 | 160 |
|  | Scored | 260 | 208.4 | 4h 20m | 40 | M | 28.9 | 51.7 | 0 | 85 | 142 |
|  | Scored | 223 | 130.8 | 3h 43m | 65 | M | 28.93 | 27.4 | 0 | 33 | 93 |
|  | Scored | 397 | 65.5 | 6h 37m | 28 | M | 30.78 | 15.4 | 0 | 0 | 86 |
|  | Scored | 383.5 | 55.5 | 6h 23m | 24 | M | 23.09 | 2.5 | 0 | 1 | 14 |
|  | Scored | 324.5 | 83 | 5h 24m | 52 | F | 33.52 | 16.8 | 0 | 10 | 53 |
|  | Scored | 285.5 | 118.9 | 4h 45m | 47 | M | 28.8 | 8.2 | 0 | 0 | 36 |
|  | Scored | 385 | 101 | 6h 25m | 54 | M | 32.26 | 37.1 | 4 | 39 | 168 |
|  | Scored | 300 | 62.7 | 5h 0m | 66 | M | 23.5 | 5.0 | 0 | 1 | 27 |
|  | Scored | 360.5 | 50.5 | 6h 0m | 45 | F | 44.99 | 27.5 | 1 | 36 | 73 |
|  | Scored | 324.8 | 37.5 | 5h 24m | 40 | M | 34.89 | 89.8 | 0 | 93 | 190 |
|  | Scored | 492.2 | 9.5 | 8h 12m | - | - | 33.96 | 16.5 | 0 | 2 | 107 |
|  | Scored | 411.5 | 66.1 | 6h 51m | 39 | F | 23.98 | 5.1 | 0 | 0 | 30 |
|  | Scored | 367 | 91.2 | 6h 7m | 68 | M | 27.78 | 11.6 | 0 | 0 | 78 |
|  | Scored | 372 | 115.3 | 6h 12m | 63 | M | 22.92 | 22.4 | 0 | 37 | 113 |
|  | Scored | 240.5 | 83.2 | 4h 0m | 67 | F | 32.73 | 71.9 | 7 | 71 | 155 |
|  | Scored | 448.5 | 59.2 | 7h 28m | 57 | F | 28.03 | 19.0 | 0 | 65 | 71 |
|  | Invalid<4h | - | - | - | 45 | M | 25.34 | 16.6 | 0 | 0 | 33 |
|  | Scored | 246.3 | 112.5 | 4h 6m | 62 | F | 26.5 | 4.6 | 0 | 0 | 19 |
|  | Scored | 480.5 | 87.2 | 8h 0m | 19 | M | 24.77 | 14.0 | 0 | 3 | 112 |
|  | Scored | 306.5 | 57.3 | 5h 6m | 24 | M | 21.93 | 4.7 | 1 | 3 | 28 |
|  | Scored | 416.5 | 47.5 | 6h 56m | 61 | F | 33.05 | 29.0 | 0 | 12 | 131 |
|  | Scored | 416.5 | 106.7 | 6h 56m | 31 | M | 31.16 | 33.7 | 0 | 0 | 231 |
|  | Scored | 439 | 78.4 | 7h 19m | 34 | F | 25.66 | 0.5 | 0 | 0 | 4 |
|  | Scored | 398.5 | 28.6 | 6h 38m | 46 | F | 36.44 | 11.7 | 0 | 1 | 50 |
|  | Invalid | - | - | - | 33 | M | 29.63 | 62.4 | 0 | 68 | 194 |
|  | Invalid | - | - | - | 33 | M | 26.88 | 31.8 | 0 | 81 | 73 |
|  | Invalid<4h | - | - | - | 26 | M | 36.03 | 1.6 | 0 | 0 | 2 |
